# Immune Checkpoint Ligand Reverse Signaling: Looking Back to Go Forward in Cancer Therapy

**DOI:** 10.3390/cancers11050624

**Published:** 2019-05-04

**Authors:** Daniele Lecis, Sabina Sangaletti, Mario P. Colombo, Claudia Chiodoni

**Affiliations:** Molecular Immunology Unit, Research Department, Fondazione IRCCS Istituto Nazionale dei Tumori, 20133 Milan, Italy; daniele.lecis@istitutotumori.mi.it (D.L.); sabina.sangaletti@istitutotumori.mi.it (S.S.); mariopaolo.colombo@istitutotumori.mi.it (M.P.C.)

**Keywords:** immune checkpoint, immunotherapy, PD-1/PD-L1, reverse signaling

## Abstract

The so-called immune checkpoints are pathways that regulate the timing and intensity of the immune response to avoid an excessive reaction and to protect the host from autoimmunity. Immune checkpoint inhibitors (ICIs) are designed to target the negative regulatory pathways of T cells, and they have been shown to restore anti-tumor immune functions and achieve considerable clinical results. Indeed, several clinical trials have reported durable clinical response in different tumor types, such as melanoma, renal cell carcinoma (RCC) and non-small cell lung cancer (NSCLC). Nonetheless, after the initial enthusiasm, it is now evident that the majority of patients do not benefit from ICIs, due to innate or acquired tumor resistance. It is therefore mandatory to find ways to identify those patients who will respond and to find ways to induce response in those who at present do not benefit from ICIs. In this regard, the expression of programmed death ligand 1 (PD-L1) on neoplastic cells was the first, and most obvious, biomarker exploited to predict the activity of anti-programmed death 1 (PD-1) and/or anti-PD-L1 antibodies. As expected, a correlation was confirmed between the levels of PD-L1 and the efficacy of anti-PD-1 therapy in melanoma, NSCLC and RCC. However, further results from clinical trials showed that some patients display a clinical response regardless of tumor cell PD-L1 expression levels, while others do not benefit from ICI treatment despite the expression of PD-L1 on neoplastic elements. These findings strongly support the notion that other factors may be relevant for the efficacy of ICI-based treatment regimens. Furthermore, although the current dogma indicates that the PD-1/PD-L1 axis exerts its regulatory effects via the signal transduced in PD-1-expressing T cells, recent evidence suggests that a reverse signaling may also exist downstream of PD-L1 in both tumor and immune cells. The reverse signaling of PD-L1, but also of other immune checkpoints, might contribute to the pro-tumoral/immune suppressive environment associated with tumor development and progression. Clarifying this aspect could facilitate the prediction of patients’ clinical outcomes, which are so far unpredictable and result in response, resistance or even hyper-progressive disease in some cases.

## 1. Introduction

The tumor microenvironment (TME) represents the main site where neoplastic and immune system cells interact. This cross-talk contributes to tumor progression, dissemination and metastasis [1]. The in-depth study of the TME has revealed some of the basis for such “adverse” interaction, identifying the different immune cell subsets present at the tumor site that are responsible for the local, and likely systemic, immune suppressive state. These cells include CD4 FOXP3+ regulatory T cells (Tregs), tumor-associated macrophages (TAMs) and myeloid-derived suppressor cells (MDSCs); all these subsets are capable of inhibiting effector T cell anti-tumor immune response, although with different mechanisms. The identification of these suppressive immune cell subsets and of their mechanisms of action suggests that the reversing of their suppressive activities could restore an efficacious anti-tumor immune response. Among the several inhibitory pathways that could be responsible for T cell unresponsiveness, the so-called immune checkpoints, such as Cytotoxic T-Lymphocyte Antigen 4 (CTLA-4)/B7s and the programmed death 1/programmed death ligand 1 (PD1/PD-L1) axes, are the most widely studied. Pioneering work from Jim Allison and co-workers in the mid-1990s showed that antibodies blocking CTLA-4 were able to enhance the anti-tumor immune response in mice, resulting in complete tumor rejection and long-lasting immunity [2]. This result, and subsequent preclinical studies, fostered the idea that the releasing of the “brakes” of the immune system could increase anti-tumor immunity, thus paving the way for the clinical development of CTLA-4 antibodies and starting the new era of immune checkpoint therapy. The other most studied T-cell inhibitory pathway is constituted by the PD-1/PD-L1 axis. As for CTLA-4, preclinical studies in mouse tumor models demonstrated the potential therapeutic efficacy of anti-PD-1 and anti-PD-L1 antibodies. The inhibition of negative immune checkpoints, such as CTLA-4 and the PD-1 axis, is now at the forefront of immunotherapeutic approaches for several types of cancers. Indeed, their blockade has elicited durable anti-tumor responses and long-term remission in a number of patients with different types of neoplasia [3].

Despite the great enthusiasm for the first positive results, overall, the majority of patients do not benefit from therapy with immune checkpoint inhibitors (ICIs). Resistance to ICIs could be either innate in the case of non-responders, or acquired, if following an initial objective response. Resistance can be further subdivided into intrinsic, if elicited by the tumor itself, or extrinsic, when depending on the interaction with different cells composing the TME. Additionally, environmental host factors such as microbiota, diet, hormone levels and metabolisms can further contribute to the failure of ICI therapy [4]. Besides resistance, the recent occasional observation that a few cancer patients treated with anti-PD-1/PD-L1 mAbs face a rapid worsening of the disease raised the issue of whether ICIs in some cases could be even detrimental [5,6]. Defining the mechanisms underlying the different types of resistance and those of potential hyper-progression would allow the identification of patients to be treated with the highest possibility of benefitting from ICI therapy. This review, beyond summarizing the major features of immune checkpoints, details the new evidence supporting the existence of a reverse signaling cascade mediated by immune checkpoint ligands, in particular PD-L1, and discusses whether this signaling may contribute to the final outcome of ICI treatment and/or potentially explain unexpected clinical results.

## 2. One- and Two-Way Immune Checkpoints

The immune system defends the organism from pathogens as well as from malignant cells; however, at the same time, it needs to maintain tolerance toward the self. A finely regulated T cell activation is therefore pivotal to inducing protective immunity as well as to preventing auto-immunity. The tuning of the specific response is regulated by the so-called immune checkpoint pathways. The initial definition of immune checkpoints referred mainly to the interaction between a receptor and its ligand leading to the suppression of T cell activities. This concept has gradually expanded to also include stimulatory interactions that enhance T cell functions. Additionally, it is now clear, at least for specific receptor/ligand pairs, that the signals are not unidirectional towards the cell that express the receptor (mainly T cells), but also involve a reverse activity toward the ligand-expressing cell, likely an antigen-presenting cell (APC).

One of the most studied two-way immune checkpoints is the CD40/CD40L axis. CD40 is mainly expressed by B lymphocytes and APCs, such as dendritic cells and macrophages, and its ligand CD40L by T cells, either activated CD4 T cells or Tregs [7]. Although this axis was initially thought to signal mono-directionally only inside CD40-expressing cells, there is now evidence showing that reverse signaling by CD40L is critical for T cell maturation and differentiation into T helper cells [8]. Moreover, it regulates the production of IL-4 by T cells upon antigen encounter [9]. Data on potential reverse signaling are also available for the CTLA-4/B7s pathway. In fact, it has been proven that the triggering by CTLA4 of its high affinity partner CD80 on dendritic cells (DCs) induces STAT3 phosphorylation and a reduction of NF-κB activity, which in turn lead to the down-modulation of CD80 and CD86, and likely to the development of tolerogenic DCs [10]. Also, for the OX40/OX40L axis, there is evidence for reverse signaling in OX40L-expressing cells. Accordingly, the engagement of OX40L, expressed on activated B cells, by OX40 receptor, present on activated T cells, stimulates B cell proliferation and immunoglobulin secretion [11]. OX40L triggering seems also to affect monocyte and dendritic cell differentiation by increasing the expression of costimulatory molecules such as CD40, CD80 and CD86 and their production of TNF, IL-1, IL-6 and IL-12 [12]. Finally, the engagement of OX40L expressed on mast cells (MCs) has been shown to influence the IgE-dependent MC degranulation [13].

## 3. Starting from the Beginning: The CTLA-4-CD28/B7s Axis

Despite the extension of the definition of “immune checkpoints”, it is a common habit to consider archetypical immune checkpoints as the only the inhibitory ones and particularly the two most studied receptor/ligand pairs, i.e., CTLA-4/B7s and PD-1/PD-L1. The particular attention paid to these two axes is clearly fueled by the success of their blockade in clinical practice. The first and best-characterized immune checkpoint pathway is represented by the costimulatory receptor CD28 and the co-inhibitory receptor CTLA-4, which both interact with their shared ligands CD80 (B7-1) and CD86 (B7-2). The binding of CD28 to CD80 or CD86 represents the second signal required for efficient T cell stimulation, which is triggered through the interaction of T cell receptor (TCR) with the peptide-MHC-I complex. CTLA-4 is rapidly up-regulated upon TCR engagement and it competes with CD28 for CD80 and CD86, having a greater affinity and avidity [14,15]. Therefore, the presence of CTLA-4 at the immunological synapse dampens the strength of CD28 downstream signals, which are mediated by AKT and Phosphoinositide 3-kinase (PI3K) [16,17]. Moreover, through its cytoplasmatic tail and depending on its phosphorylation status, CD28 interacts with a number of proteins, which eventually lead to the activation of AP-1, NFAT and NF-kB transcription factors (for a comprehensive review on CD28 signal transduction, refer to [18]). In this way, CD28 together with TCR signaling causes the activation of a complex transcriptional program in T cells, which is fundamental for the production of IL-2 and the up-regulation of interferon (IFN)-γ [16]. High levels of CTLA-4 are associated with reduced activation of T cells at the secondary lymphoid sites, where T cells are primed, and also in peripheral tissues. Moreover, CTLA-4 expressed by Tregs has been shown to be fundamental to maintain systemic tolerance [19,20,21]. Accordingly, CTLA-4 knock-out mice develop a lethal autoreactive and hyperproliferative lymphocyte expansion, strongly supporting the immune-regulatory role of this molecule [22,23]. Of note, CTLA-4 can play both a cell-intrinsic and a cell-extrinsic role, which results in an *in cis* or *trans* [20] regulation, respectively.

## 4. The PD-1/PD-L1 Axis as the New Main Character in the Immunotherapy Field

PD-1 is expressed in an inducible fashion on activated B and T cells, while its ligands, PD-L1 and PD-L2, can be expressed on a plethora of different cell types including myeloid, epithelial and tumor cells [24]. Also, PD-L1 expression can be stimulated in a transient manner, especially in response to inflammatory cytokines such as IFN-γ. Since PD-1 ligands are expressed in several non-lymphoid tissues, the PD-1/PD-L1 axis inhibits T cell activity mostly in the periphery. Upon stimulation, PD-1 propagates an inhibitory signal through the tyrosine phosphatase SHP2 that dephosphorylates TCR signaling molecules, such as Zap70 [25], leading to the suppression of T cell activation [26]. Recent work demonstrated that the co-stimulatory receptor CD28, rather than the TCR, may be a primary target for dephosphorylation by the SHP2 phosphatase after PD-1 triggering [27], suggesting that different mechanisms may collaborate to regulate effector T cell function and response to anti-PD-L1/PD-1 therapy.

Activated T cells thus express PD-1, which is maintained together with other specific molecules, such as Tim3 and LAG-3, in exhausted T cells. In the latter subsets of cells, PD-1 also regulates metabolism by reducing glycolysis while simultaneously favoring fatty acid oxidation and lipid catabolism [28,29]. As for CTLA-4, the proof that PD-1 plays a crucial role in controlling tolerance was confirmed by generating knock-out mice which developed severe strain-dependent autoimmunity [30,31], even if less harmful than that observed in CTLA-4 knock-out mice. The latter observation supports the idea that CTLA-4 and PD-1 take part to the tolerance process in different stages, the former playing a very early function already in the lymphoid organs, and the latter having a role at later stages in the periphery.

## 5. Immune Checkpoint Blockade: A Great Clinical Success with a Few “Buts”

The blockage of immune checkpoints has been shown to induce durable responses in several different types of cancer. Ipilimumab, an anti-CTLA-4 antibody, was the first ICI to be FDA-approved in 2011 for the treatment of metastatic melanoma. Thereafter, five other immune checkpoint-targeted therapies have been approved, all directed against PD-1 or PD-L1, for the treatment of melanoma, non-small cell lung cancer (NSCLC), renal cell carcinoma (RCC) and a number of other tumor types, in monotherapy and combinatorial regiments. Unfortunately, only a subset of patients reached a response, making it mandatory to identify novel predictive markers of response to treat only patients who would benefit from this type of therapy [32]. The first markers to be exploited were PD-L1 expression levels on cancer cells [3,33,34,35] and the presence of tumor-infiltrating lymphocytes (TILs). In fact, while patients with tumors expressing higher levels of PD-L1 generally have a poorer prognosis [36,37,38], at the same time, they were shown to benefit the most from ICI treatment, even though this evidence is still controversial [39,40,41]. This discrepancy suggests that the prognostic role of PD-L1 expression could be cancer subtype-specific [33]. Intuitively, the presence of immune cells in close proximity to tumor cells would favor the efficacy of ICIs and, accordingly, the levels of immune system infiltrating tumors were shown to be associated with pathological response [42,43,44]. Low levels of innate [45,46] and high infiltration of adaptive immune [43,44] cells were shown to predict a better response. Nonetheless, different subsets, even of the same immune cell type, could result in different outcomes when stimulated with anti-PD-1 therapy [47,48]. Obviously, different biomarkers can also be linked to each other, and this is particularly true for PD-L1 expression and immune infiltration. In fact, cancer cells up-regulate the expression levels of PD-L1 in response to INF-γ released from infiltrating T cells and in this way acquire immune resistance [49], while becoming more likely responsive to therapy directed against the PD-1/PD-L1 axis. This implies that both parameters should be considered simultaneously in order to predict response to treatment [50]. Moreover, a number of works have shown that not only PD-L1 produced by cancer cells is predictive of response to ICIs, but also the one expressed on immune cells plays a crucial role in determining the final outcome [51,52]. As already mentioned, the presence of infiltrating leukocytes can induce the expression of PD-L1 by cancer cells in an IFN-γ-dependent manner and hence the expression of genes stimulated by this cytokine has been proposed as a marker of response to ICI treatment [53]. Additionally, cancer cells can also express PD-L1 because of specific oncogenic signaling, such as RAS, which up-regulates its expression through a mechanism involving increases in PD-L1 mRNA stability [54], or the EML4-ALK fusion gene and mutant EGFR, which up-regulate PD-L1 by activating PI3K-AKT and MEK-ERK signaling pathways in NSCLC [55].

The presence of neo-antigens would favor the efficacy of therapies aiming at restoring a functional immune anti-tumor activity [56] and this is true when considering both anti-PD-1 and anti-CTLA-4 antibodies, though with different mechanisms of action—i.e., anti-PD-1 being active at the tumor site, and anti-CTLA-4 at the periphery in the lymphoid organs where T cell priming takes place. CTLA-4 is also thought to kill Treg via ADCC at tumor site and shape vasculature in the TME [57]. Accordingly, there is growing interest for the study of tumor mutational burden (TMB) as a predictive marker and for the employment of ICIs in tumors with genomic instability, such as tumors with microsatellite instability (MSI) due to DNA mismatch repair deficiency [58]. Notably, TMB cannot be considered a perfect surrogate of immunogenicity and, supporting this view, not all tumors characterized by high TMB were shown to respond to ICIs. Uncertainty is also linked to the different cut-off used to score TMB. The recent possibility of TMB detection in blood, without the need of adequate tumor tissue for molecular testing, could greatly simplify this analysis, although the prediction is so far limited to progression-free survival [59].

## 6. When the Treatment Makes Things Worse: The Strange Case of Hyper-Progression

About 10–30% of patients treated with ICIs in monotherapy experience a durable anti-tumor effect significantly longer compared to standard treatment options [60], and with limited toxicity, often ascribable to immune-related adverse events. Nonetheless, most patients do not benefit from this kind of treatment due to intrinsic or acquired resistance, which can stem from cell autonomous or non-autonomous mechanisms [61]. Interestingly, in a limited subset of patients, treatment with ICIs initially seems to stimulate cancer growth, which however is suddenly followed by tumor shrinkage [62]. This phenomenon is referred to as “pseudo-progression” and interests only a minor percentage of treated patients (<10%). Nonetheless, a slightly larger group of patients face a rapid worsening of the disease upon treatment with ICIs, defined as hyper-progressive disease (HPD), with a median overall survival of about 3 months [60]. This phenomenon does not seem to be associated with the type of tumor, and it has been observed in patients treated with both PD-1 and PD-L1 inhibitors [5,63], but, interestingly, not with anti-CTLA-4 antibodies. Works describing HPD have reported variable percentages of patients facing this event, and this discrepancy is strongly biased by the method used to define HPD, and to distinguish it from the natural progression of the disease.

The underlying mechanism of HPD is still debated and several hypotheses have been formulated to explain this phenomenon. In particular, ICIs could promote the activation and proliferation of T suppressor cells, as Tregs, which, in the case of chronic inflammation (e.g., infection or cancer), minimize potential detrimental effects of the immune system without a complete block of inhibition of its activity, through a mechanism called contra-suppression [64,65]. Consequently, treatment with anti-PD-1/PD-L1 antibodies could promote the proliferation of tumor-specific Tregs, favoring an immune-suppressed TME. ICIs could also negatively influence the activity of specific T cell subsets such as T helper 2 [66] or follicular Tregs [67]. Finally, a recent work suggested that TAMs could be reprogrammed upon Fc receptor engagement by ICIs, and ultimately could be responsible for the induction of HPD [6]. All the described mechanisms are clearly not mutually exclusive and could occur simultaneously by promoting HPD to a variable extent. Incontrovertible biomarkers are hence necessary to select the patient subsets that would benefit by ICI treatment and, even more urgent, necessary to spare patients who might develop HPD.

### 6.1. PD-L1 Reverse Signaling in Tumor Cells

**Figure 1:**
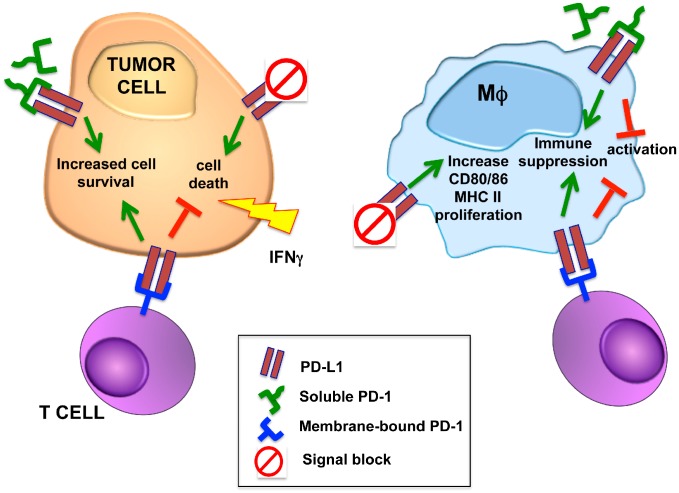
PD-L1 reverse signaling in tumor cells and macrophages. Besides the effects mediated by PD-L1 triggering of PD-1 expressed by T cells, recent evidence supports the existence of a reverse signaling in PD-L1 expressing cells, either tumor cells or macrophages. In the case of neoplastic cells, it has been shown that the short intra-cytoplasmatic tail of the PD-L1 molecule contains some non-classical signal transduction motifs that mediate protection from IFNγ-induced cell death [52]. Similarly, PD-L1 reverse signaling, mediated by the binding of either cell-bound or soluble PD-1, has been demonstrated to support the proliferation and survival of classical Hodgkin lymphoma cells [68]. Differently from neoplastic cells, in macrophages, a PD-L1 cell intrinsic pathway seems to stimulate a constitutive inhibitory signal that, when interrupted, induces the up-regulation of activation markers, such as CD80, CD86 and MHC II molecules, and activates an anti-tumor phenotype in tumor-associated macrophages (TAMs) [69].

The inhibitory signals transduced by PD-1 in T cells upon PD-L1 triggering have been extensively characterized. However, the PD-1-independent activities of PD-L1 are less characterized and it is now hypothesized that PD-L1 could also be able to propagate signals inside the cell on which it is expressed (Figure 1). This reverse signaling is still poorly characterized and could contribute to the final outcome of ICI treatment.

In fact, recent evidence indicates that PD-L1 can activate, also in the absence of PD-1, cell-intrinsic signals in tumor cells that induce proliferation and survival, while inhibiting autophagy and mTOR activity [70]. Since PD-L1 is a transmembrane protein with a very short intracytoplasmic domain also lacking the canonical sequence motifs capable of transducing a signal, the general idea was that PD-L1 activity is exerted only through the triggering of a signal in PD-1-expressing cells. However, a recent paper demonstrated that PD-L1 expressed by tumor cells delivers a cell-intrinsic signal that protects them from IFN cytotoxicity and that the abrogation of PD-L1 expression, or its blockade through an antibody, sensitizes cancer cells to IFN cytotoxicity through a STAT3/caspase-7-mediated pathway (Figure 1 and [71]). The authors identified in its short cytoplasmic tail some non-classical signal transduction motifs that are essential for such a protective function. This work clearly demonstrated that, independently from its inhibitory activity on T cells, PD-L1 also provides cancer cells with a protective shield to counteract IFN-induced toxicity, ultimately contributing to tumor growth and progression. In addition to its interaction with PD-1, PD-L1 can also bind to CD80, and it has been shown that upon such interaction, it delivers inhibitory signals to activated T cells, resulting in reduced proliferation and cytokine production [72].

In the last few years, the evidence for a PD-L1 reverse signaling has grown. Chen and colleagues showed a tumor cell autonomous role of PD-L1 signaling in promoting epithelial to mesenchymal transition (EMT) in human esophageal cancer [73]. By manipulating PD-L1 expression in an esophageal cancer cell line, by either ablation, overexpression or mutation of its cytoplasmic tail, they demonstrated that PD-L1 has a role in the cellular viability, migration and EMT phenotype of tumor cells [73].

PD-L1 expression on tumor cells has also been associated with a stem-like phenotype, in particular in triple negative breast cancer (BC) [74]. Analyses of large datasets of BC patients showed a significant correlation between its expression and a stemness score [74,75]. Besides the expected role in sparing PD-L1-expressing cancer stem cells (CSCs) from T cell immune attack, there is evidence that PD-L1 can also directly affect the expression of stemness markers in CSCs. By using PD-L1 knockdown experiments, Almozyan and colleagues demonstrated that PD-L1 has a critical role in maintaining the expression of OCT-4, Nanog and BMI1, and that its down-regulation reduces the self-renewal capability of breast CSCs both in vitro and in vivo [75].

PD-L1 intracellular signaling has been shown not only in solid tumors, but also in the case of hematological malignancies. Refractory and relapsed classical Hodgkin lymphomas (cHL) respond quite efficaciously to treatment with PD-1 blocking Ab, suggesting that the PD-1/PD-L1 signaling pathway is relevant for disease progression. Jalali and co-workers reported that the direct engagement of PD-L1 with an agonist Ab on HL cell lines results in increased survival and proliferation, in addition to reducing apoptosis (Figure 1 and [68]). They detected in the serum of cHL patients a higher level of soluble PD-1, which likely triggers PD-L1 on HL cells, hence favoring their proliferation and survival through the activation of the MAPK pathway. Therefore, they hypothesized that a reverse signaling through PD-L1 may represent a potential mechanism contributing to cHL progression [68]. This observation could contribute to the understanding of the good response of cHL patients to anti-PD-1 Ab treatment.

### 6.2. PD-L1 Reverse Signaling in Immune Cells

Besides tumor cells, PD-L1, as well as PD-L2, is also expressed by cells of the immune system, in particular by those of the myelo-monocytic lineage such as monocytes, macrophages, dendritic cells and MDSCs, and its expression is often up-regulated within the tumor milieu. Of note in this lineage, the acquisition of PD-L1 expression has been reported to occur very early in differentiation at the stages of Lin−/Sca−/Kit+, common myeloid progenitors and granulocyte-monocyte progenitors [76]. The mechanisms responsible for the up-regulation of PD-L1 on myeloid cells within the TME have been partly elucidated and were shown to depend on the p-STAT1-IRF1 axis [77], on IFN receptor type I (IFNAR1) expression [78] and on the COX2/mPGES1/PGE2 pathway [79].

The hypothesis that the contribution of PD-L1 expressed by tumor cells and by myeloid cells in mediating immune suppression of anti-tumor T cell response is non-redundant was elegantly demonstrated in a recent work by Lau and coworkers [52]. In mouse tumor models, they showed that only the inhibition of both signals could lead to efficient tumor regression with almost complete prevention of tumor escape [52]. Contrarily, the prevention of PD-L1 signaling only on one compartment at a time reduced tumor growth by 50%. These results suggest that PD-L1 expression by infiltrating myeloid cells provides a partially compensatory source of PD-1 ligand in the case of PD-L1 negative tumors, which is sufficient to dampen T cell response. The reduced growth of PD-L1-expressing tumors in PD-L1-deficient hosts indicates that its expression by infiltrating myeloid cells still plays a role in negatively regulating T cell response, despite the high expression of PD-L1 by tumor cells [52].

As for tumor cells, the possibility that PD-L1 could transduce intrinsic signals also in cells of the myeloid lineage has recently been investigated, at least in macrophages. By employing in vitro and in vivo models, Hartley and colleagues studied PD-L1 signaling on TAMs function (Figure 1 and [69]). In vitro CSF-1-generated murine bone marrow- and human monocyte-derived macrophages treated with anti-PD-L1 Ab showed a higher proliferation rate than control-Ab-treated cells. Moreover, they were more activated, expressing higher levels of CD80 and MHC-II molecules, and producing more TNF and IL-12. Interestingly, when testing the cognate ligands for PD-L1, they found that CD80 was more efficient than soluble PD-1 in modifying the macrophage phenotype. Taking advantage of PD-L1 knock-out (KO) macrophages, they demonstrated that PD-L1 triggers a constitutive and negative signal in macrophages, and that the binding of PD-L1 with antibodies inhibits this negative signaling, therefore resulting in proliferation, survival and activation. A deeper investigation of these mechanisms indicated that PD-L1 constitutively propagates its signal by blocking the mTOR pathway cascade. In vivo experiments performed in immune-deficient mice demonstrated a T cell-independent therapeutic activity of anti-PD-L1 Ab, likely mediated by its effects on the phenotype of TAMs, which, upon treatment, are more activated and likely anti-tumor [69]. Although still not directly proven, a similar mechanism of PD-L1 reverse signaling can likely also occur in MDSCs. Ballbach and colleagues recently demonstrated that the blocking of PD-L1 partially impairs MDSC-mediated T cell suppression [80]. This effect is most likely the consequence of targeting PD-L1, which prevents the triggering of PD-1 on T cells. Nonetheless, so far, there is no evidence of the existence of a PD-L1 cell-intrinsic signaling that could affect the "functional" state of myeloid cells independently from the PD-1-mediated effects on T cell function and proliferation. As done for macrophages [69], a more detailed investigation on the intracellular signaling pathways activated, or inhibited, by PD-L1 triggering on MDSCs, with either antibodies or soluble PD-1, could provide useful insights and potentially drive the choice of the therapeutic antibody to be used, i.e., anti-PD-1 versus anti-PD-L1 in patients showing a significant expansion of MDSCs. Table 1 summarizes the recent pieces of evidence for PD-L1 reverse signaling in different cell types.

## 7. Conclusions

The advent of ICIs has profoundly changed the concept of immunotherapy and brought novel and promising opportunities in cancer treatment. In fact, the targeting of immune checkpoints in many tumor types resulted in stable anti-tumor effects, which were shown to be more durable compared to traditional chemotherapy and less toxic. Nonetheless, only a subset of patients benefits from this type of therapy, making it mandatory to identify the mechanisms underlying intrinsic and extrinsic resistance in order to overcome it, as well as those patients who may experience HPD. Moreover, there is increasing evidence that ligands of the immune checkpoint pathways, in particular PD-L1, could also trigger a receptor-independent signal inside the cells in which they are expressed and that these signals could be different depending on the specific cell types. Therefore, it is still crucial to identify biomarkers that could predict these phenomena and to develop novel preclinical models suitable to investigate the underlining molecular mechanisms.

## Figures and Tables

**Table 1:** Evidence of PD-L1 reverse signaling.

Cell Types	Biological Effects	Experimental Setting	Reference
Mouse ovarian cancer (ID8) melanoma (B16)	PD-L1 down-modulation enhanced autophagy, reduced mTORC1 activity and reduced tumor growth and metastasis	RNA interference	[70]
B16 melanoma (CT26 colorectal and 4T1 breast cancer)	PD-L1 signaling protects cancer cells from interferon (IFN) cytotoxicity and accelerates tumor progression	CRISPR-Cas9; mutations in intracellular domains	[71]
T cells	Inhibitory interaction between B7-1 (CD80) and PD-L1 that affects T cell activation and cytokine production	Cd28^−/−^, Ctla4^−/−^, Cd274^−/−^ cells; in vitro binding assays with Ig fusion proteins	[72]
Human esophageal cancer (Eca-109 cell line)	PD-L1 expression promoted cell viability, migration and epithelial to mesenchymal transition (EMT) phenotype	RNA interference and over-expression	[73]
Breast cancer (MDA-MB-231 cell line)	PD-L1 expression necessary for expression of OCT-4A, Nanog and the stemness factor, BMI1 in cancer stem cells	PD-L1 knock-down by shRNA and ectopic expression	[75]
Classical Hodgkin lymphoma (HL cell lines)	Stimulation of the HL cell lines with PD-L1 antibody increases cell survival and proliferation and reduces apoptosis	In vitro stimulation with agonist PD-L1 Ab	[68]
Bone marrow-derived macrophages, tumor-associated macrophages	PD-L1 signal block activates macrophages (CD80, MHC II up-regulation, increased IL-12 and TNF production); PD-L1 signals constitutively inhibit mTOR pathway signaling	In vitro Ab treatment, sPD-1 and sCD80 stimulation; PD-L1 KO macrophages; in vivo effect on tumor growth of B16 melanoma and PyMT breast tumors and macrophage phenotype	[69]

PD-L1: programmed death ligand 1; TORC1: Target of rapamycin complex 1; CTLA-4: cytototoxic T lymphocyte antigen 4; CRISPR: Clustered Regularly Interspaced Short Palindromic Repeats; OCT-4: octamer-binding transcription factor 4; BMI1: B-cell-specific Moloney murine leukemia virus integration site 1; shRNA: short hairpin RNA; HL: Hodgkin Lymphoma; TNF: tumor necrosis factor; PyMT: polyoma middle T.
